# Contrasting genetic association of *IL2RA *with SLE and ANCA – associated vasculitis

**DOI:** 10.1186/1471-2350-10-22

**Published:** 2009-03-05

**Authors:** Edward J Carr, Menna R Clatworthy, Christopher E Lowe, John A Todd, Andrew Wong, Timothy J Vyse, Lavanya Kamesh, Richard A Watts, Paul A Lyons, Kenneth GC Smith

**Affiliations:** 1 Cambridge Institute for Medical Research, University of Cambridge School of Clinical Medicine, Addenbrooke's Hospital, Hills Road, Cambridge, CB2 0XY, UK; 2 Department of Medicine, University of Cambridge School of Clinical Medicine, Addenbrooke's Hospital, Hills Road, Cambridge, CB2 0XY, UK; 3 Juvenile Diabetes Research Foundation/Wellcome Trust Diabetes and Inflammation Laboratory, Department of Medical Genetics, University of Cambridge School of Clinical Medicine, Addenbrooke's Hospital, Hills Road, Cambridge, CB2 0XY, UK; 4 Imperial College, Molecular Genetics and Rheumatology Section, Hammersmith Hospital, Du Cane Road, London, W12 0NN, UK; 5 Division of Infection and Immunity, Medical School, University of Birmingham, Birmingham, B15 2TT, UK; 6 School of Medicine, Health Policy and Practice, University of East Anglia, Norwich, NR4 7TJ, UK

## Abstract

**Background:**

Autoimmune diseases are complex and have genetic and environmental susceptibility factors. The objective was to test the genetic association of systemic lupus erythematosus (SLE) and anti-neutrophil cytoplasmic antibody (ANCA) – associated systemic vasculitis (AAV) with SNPs in the *IL2RA *region and to correlate genotype with serum levels of IL-2RA.

**Methods:**

Using a cohort of over 700 AAV patients, two SLE case-control studies and an SLE trio collection (totalling over 1000 SLE patients), and a TaqMan genotyping approach, we tested 3 SNPs in the IL2RA locus, rs11594656, rs2104286 & rs41295061, each with a prior association with autoimmune disease; rs11594656 and rs41295061 with type 1 diabetes (T1D) and rs2104286 with multiple sclerosis (MS) and T1D.

**Results:**

We show that SLE is associated with rs11594656 (*P *= 3.87 × 10^-7^) and there is some evidence of association of rs41295061 with AAV (*P *= 0.0122), which both have prior association with T1D. rs2104286, an MS and T1D – associated SNP in the *IL2RA *locus, is not associated with either SLE or AAV.

**Conclusion:**

We have confirmed a previous suggestion that the *IL2RA *locus is associated with SLE and showed some evidence of association with AAV. Soluble IL-2RA concentrations correlate with rs11594656 genotype in quiescent disease in both AAV and SLE. Differential association of autoimmune diseases and SNPs within the *IL2RA *locus suggests that the *IL2RA *pathway may prove to play differing, as yet undefined, roles in each disease.

## Background

Most autoimmune diseases are polygenic, with genetic susceptibility being conferred by common variants at a number of loci. It is evident that some susceptibility loci are shared between autoimmune diseases. One locus that is associated with several diseases including T1D, MS and Graves' disease, is *IL2RA *[1-4].

The IL-2 pathway is critical for the maintenance of peripheral tolerance. Activated T cells and regulatory T cells express IL-2RA, the high affinity IL-2 receptor (or CD25). IL-2RA expression has also been reported on activated B cells, activated monocytes, and NK cells [5]. IL-2 signalling supports the division and survival of effector T cells, plays a role in activation-induced cell death and is vital to regulatory T cell homeostasis [6]. Its physiological role on other IL-2RA expressing cell types, however, is unclear. One human patient, homozygous for a 4 base pair exonic deletion in *IL2RA*, lacking a functional IL-2RA demonstrated increased susceptibility to viral, bacterial and fungal infections, but unlike *Il2ra*-deficient mice did not develop overt autoimmune disease [7]. Thus, the IL-2 pathway has a profound effect on the T cell immune response, contributing to both activation and regulation depending on the immunological context.

In T1D, two disease-associated SNPs [rs11594656 and rs41295061] are required to explain association with this region of chromosome 10p15, indicating that there are multiple functional alleles of the *IL2RA *locus, presumably altering expression of the receptor [1,2]. In addition, associations have been established with multiple sclerosis (MS) [rs2104286] [3], and suggested with Graves' disease [4]. The SNP, rs2104286, has also been associated recently with T1D, representing a third independent effect for the *IL2RA *region in this autoimmune disease [8]. In systemic lupus erythematosus (SLE), SNPs without any prior autoimmune associations [rs7072793, rs4147539, rs7090530 and rs12251307] were examined in a candidate gene analysis of *IL2RA*, providing weak evidence for association [9]. Soluble IL-2RA (sIL-2RA) is shed from human T cells following *in vitro *activation in response to a variety of polyclonal stimuli [5]. Significantly elevated serum sIL-2RA has been described in infection, malignancy and in autoimmune disease, including T1D, rheumatoid arthritis, SLE and Wegener's granulomatosis [5,10], although the functional implications of this remain unclear. Genotype at rs11594656 and rs41295061 [2], and most recently with rs2104286 [8], has been shown to correlate with serum sIL-2RA concentrations. We therefore genotyped autoimmune disease-prone variants of the *IL2RA *region (rs11594656, rs41595061 and rs2104286) in two distinct autoimmune diseases associated with soluble IL-2RA elevation, using three independent SLE cohorts and one AAV cohort. SLE is a polygenic multi-system disease characterised by immune complex formation and deposition, frequently associated with anti-dsDNA antibodies and complement dysregulation. The clinical features vary between patients, both in severity and system involvement, and include joint, skin, renal and neurological manifestations. AAV can be divided into three clinical syndromes (Wegener's granulomatosis, microscopic polyangiitis and Churg-Strauss syndrome). All are characterised by inflammation of medium to small calibre blood vessels, and the presence of anti-neutrophil cytoplasmic antibody (ANCA). The clinical presentation of AAV is variable between patients. Severe manifestations include acute glomerulonephritis and granulomatous inflammation of the upper and lower respiratory tract. We also assessed serum titres of sIL-2RA in both diseases.

## Methods

### AAV

The AAV cohort (n = 744) comprises subjects from four sources, all meeting the Chapel Hill diagnostic criteria [11]:

1. The MRC/Kidney Research UK National DNA Bank for Glomerulonephritis (KRUK-AAV). Individuals were between the ages of 18 and 70 years, were ANCA seropositive, and had biopsy-proven necrotizing glomerulonephritis.

2. The UK vasculitis cohort 2 was recruited from 9 centres in the UK and comprised patients seropositive for ANCA and/or with histological evidence of small vessel vasculitis.

3. Patients recruited from the University of Birmingham. All individuals were ANCA seropositive with firm clinical and/or histological evidence of vasculitis.

4. The Lupus and Vasculitis Service, Addenbrooke's Hospital, Cambridge. All individuals were ANCA seropositive with firm clinical and/or histological evidence of vasculitis.

### SLE

The KRUK-SLE cohort (n = 220) was obtained from the MRC/Kidney Research UK National DNA Bank for Glomerulonephritis (KRUK) and the Lupus and Vasculitis Service, Addenbrooke's Hospital, Cambridge. All individuals were between the ages of 18 and 50 years with a definite diagnosis of lupus nephritis based on biopsy and on the clinical and serological features defined by the American College of Rheumatology (ACR) [12]. The Imperial case-control cohort comprised 480 SLE patients and there were 471 trios in the Imperial family cohort (*ie *471 affected sons/daughters, 942 parents) [13]. Both Imperial cohorts used the ACR defined clinical and serological features. Since the KRUK-SLE cohort used the additional inclusion criterion of biopsy-proven renal involvement, it was analysed separately from the Imperial case-control cohort.

Genotyping was performed using TaqMan genotyping kits (Applied Biosystems) for each SNP, with fluorescence data captured using an ABI 7900HT (Applied Biosystems) after 40 cycles of PCR.

### Assessment of disease activity

Birmingham Vasculitis Activity Scores (BVAS) were calculated for AAV patients seen by the Lupus and Vasculitis Service at Addenbrooke's hospital. BVAS assesses both constitutional and organ-specific symptoms, determining whether they are new or pre-existing [14]. British Isles Lupus Assessment Group (BILAG) scores were used to assess disease activity in SLE patients seen by the Lupus and Vasculitis Service at Addenbrooke's hospital. BILAG scores assess 9 different organ systems based upon a physician's intention to treat [15]. A flare was defined when three criteria were met: 1. Worsening disease activity scores (at least 1 major or 3 minor BVAS criteria for AAV; a new BILAG score A or B in any system for SLE), 2. clinical impression of active disease and 3. an increase in immunosuppressive treatment.

### Controls

Control genotypes for 9115 individuals from the British 1958 Birth Cohort and UK Blood Service were obtained from the Juvenile Diabetes Research Foundation/Wellcome Trust Diabetes and Inflammation Laboratory [16]. This cohort is an expansion of a dataset previously shown to be appropriate for us as UK-wide controls [17]. The 1958 Birth Cohort DNA was collected as part of an ongoing study following all births in England, Scotland and Wales in one week in 1958 . For the SLE analysis, controls were split into two groups and one group used as controls for each case cohort: n = 4534 controls for the KRUK cohort and n = 4581 for the Imperial cohort. Some of these control individuals were used in the earlier T1D studies [1,2].

### ELISA

Serum soluble IL-2RA was measured in triplicate using an ELISA, according to the manufacturer's instructions (R&D Systems). Serum from both AAV and SLE patients was collected at presentation with a 'flare' (time 0, see Assessment of disease activity section) and three months later (3 months) by the Lupus and Vasculitis Service, Addenbrooke's Hospital, Cambridge.

### Statistical analysis

Statistical analysis was performed using Prism (GraphPad), and R . Case control analyses were performed using χ^2 ^tests for significance on 2 × 2 contingency tables. Odds ratios and 95% confidence intervals were calculated from the same 2 × 2 tables. The Imperial trio data was analysed using the transmission disequilibrium test (TDT). ELISA data were tested for significance using linear regression modelling or the Mann-Whitney test, as indicated in the figure legends.

This study was approved by the Cambridge Local Research Ethics Committee and by the Oversight Committee of the KRUK DNA Bank.

## Results

**Figure 1:**
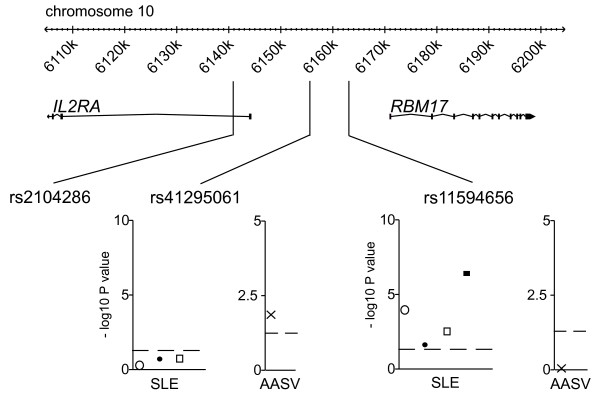
**SLE and AAV are associated with different SNPs in the *IL2RA *region**. The genomic context of rs2104286, rs41295061 and rs11594656 is shown, using the GBrowse implementation in T1DBase . All are non-coding SNPs. Negative decimal logarithms of *P *values (χ^2 ^test; 1 degree of freedom) from each cohort from SLE and AAV are plotted for rs41295061 and rs11594656 but not for rs2104286, as an association of this MS-associated SNP was not detected with SLE or AAV (table 1). Open circles represent the KRUK case-control cohort; filled circles the Imperial case-control cohort and open squares the Imperial family trios (*P *value from TDT). The filled square represents the combined *P *value for rs11594656 in SLE. Crosses are used for the AAV cohort. The dashed lines correspond to *P = *0.05.

In all three of our SLE cohorts, rs11594656 was found to be significantly associated with disease (protective minor allele, as seen in T1D), combined *P *= 3.87 × 10^-7 ^(KRUK case control *P *= 1 × 10^-4^, Imperial case control *P *= 0.0221 and Imperial family trio TDT *P *= 2.8 × 10^-3^) (Figure 1 and Table 1). Neither of the other two SNPs were associated with SLE (Figure 1 and Table 1). In AAV, in contrast, some evidence for an association was found with rs41295061 (*P *= 0.0122; minor allele odds ratio = 0.7739; 95% OR CI 0.6330 – 0.9461). The minor allele is protective as in T1D. However, neither rs2104286 or rs11594656 showed any association with AAV (Figure 1 and Table 1). At 80% power, we can detect effect sizes larger than 1.25 for rs11594656 in AAV and 1.4 for rs41295061 in SLE, across all the SLE cases and controls.

**Table 1:** Genotype data at rs11594656, rs41295061 and rs2104286 for SLE and AAV

	SLE			AAV
	KRUK cases	Imperial cases	Imperial trios	
**rs11594656 T>A**				
Genotyped cases	208	441	481	670
TT, TA, AA	148, 52, 8	276, 145, 20		382, 252, 36
Genotyped controls	4401	4449	310 *	8850
TT, TA, AA	2486, 1655, 260	2542, 1640, 267		5028, 3295, 527
MAF in cases	0.16	0.20	-	0.24
*P *value (1 d.f. test)	1 × 10^-4^	0.0221	2.8 × 10^-3^	0.7481
OR for minor allele	0.595	0.82	-	1.02
95% CI for OR	0.4570 – 0.7756	0.6931 – 0.9723	-	0.8972 – 1.163
**rs41295061 C>A**				
Genotyped cases	208	421	471	675
CC, CA, AA	172, 33, 3	330, 80, 11		569, 102, 4
Genotyped controls	4438	4498	137 *	8936
CC, CA, AA	3603, 790, 45	3603, 832, 63		7206, 1622, 108
MAF in cases	0.09	0.12	-	0.08
*P *value (1 d.f. test)	0.7187	0.1899	0.2000	0.0122
OR for minor allele	0.94	1.154	-	0.7739
95% CI for OR	0.6710 – 1.317	0.9307 – 1.438	-	0.6330 – 0.9461
**rs2104286 A>G**				
Genotyped cases	198	436	471	380
AA, AG, GG	113, 74, 11	234, 158, 44		207, 148, 25
Genotyped controls	3475	3529	353 *	7004
AA, AG, GG	1823, 1403, 249	1825, 1452, 252		3648, 2855, 501
MAF in cases	0.24	0.28	-	0.26
*P *value (1 d.f. test)	0.1760	0.7568	0.558	0.3727
OR for minor allele	1.177	0.9756	-	1.078
95% CI for OR	0.9294 – 1.490	0.8343 – 1.141	-	0.9133 – 1.273

MAF minor allele frequency; OR odds ratio; CI confidence interval; * informative transmissions. Genotype frequencies are shown for the case control cohorts.

**Figure 2:**
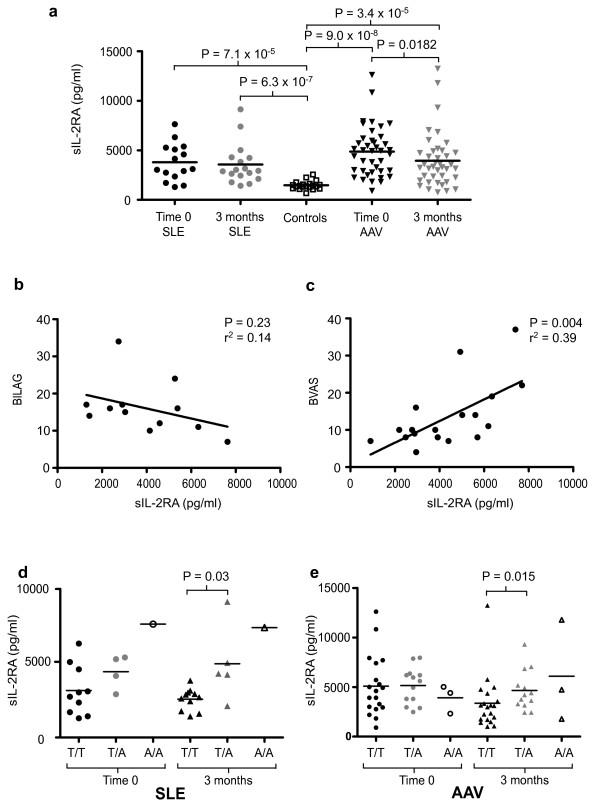
**Serum soluble IL-2RA in SLE and AAV and its relationship with disease activity and genotype at rs11594656**. (a) A comparison of SLE and AAV soluble IL-2RA (sIL-2RA) at time 0 and 3 months compared with controls, as assessed by ELISA. Each dot represents a patient. (b, c) Correlation between disease activity and sIL-2RA. (BILAG for SLE; BVAS for AAV; see supplementary information). r^2 ^and *P *values from linear regression modelling are shown. (d, e) sIL-2RA levels stratified by genotype at rs11594656 at both time 0 and 3 months for SLE (d) and AAV (e). *P *values for panels (a), (d) & (e) were generated using the Mann-Whitney test.

In both SLE and AAV, sIL-2RA concentrations were significantly higher than controls (Figure 2a). In SLE, disease activity as assessed by BILAG score did not show a significant correlation with sIL-2RA concentrations (Figure 2b), despite previous reports of a correlation between sIL-2RA concentrations and disease activity [5]. However, in AAV, disease activity as assessed by BVAS showed a positive correlation with sIL-2RA concentration (Figure 2c, [10]). Three months after commencement of treatment, when both diseases were considerably less active (mean BILAG decreased from 16.1 to 4.7 and mean BVAS from 13.9 to 4.4), sIL-2RA concentrations correlated with the SLE associated rs11594656 genotype (Figures 2d &2e). In active AAV this correlation no longer held true (figure 2e).

## Discussion

We have shown a novel *IL2RA *association with AAV and confirm the *IL2RA *locus association with SLE. This brings the number of autoimmune diseases associated with this gene to five, the others being T1D, MS and Graves' disease [1-4]. An association of SLE with *IL2RA *(rs7072793, *P *= 0.01) was generated by candidate gene re-analysis of genome-wide association data from 720 female patients [9]. The present study uses 1130 SLE genotypes, of mixed gender, in three independent cohorts, and demonstrates a strong association with the *IL2RA *region (rs11594656, *P *= 3.87 × 10^-7^). Owing to strong co-segregation of these disorders in cases and families, susceptibility loci can be shared across different autoimmune diseases. Several examples at the gene-specific level are known, including *CTLA-4 *[18] and *PTPN22 *[19]. Our study underlines that this sharing can occur at the single gene level for *IL2RA *for T1D and SLE, two diseases in which co-segregation is less well recognised.

Our findings demonstrate that the association between *IL2RA *and autoimmune disease is not a simple one. SLE is associated with rs11594656, but not rs41295061 and in AAV there is some evidence for an association with rs41295061 but not with rs11594656. Moreover, in T1D, all three SNPs are associated with disease, but the strength of association varies with each (rs41295061, *P = *6.43 × 10^-25^; rs2104286, *P *= 1.27 × 10^-13^; rs11594656, *P *= *3*.37 × 10^-6^) [2,8]. In MS, whilst rs2104286 is associated with disease [3], rs11594656 or rs41295061 are not (rs11594656, *P *= 0.293; rs41295061, *P *= 0.625) [8].

There are differences at the functional level as well, with rs11594656 genotype correlated with sIL-2RA levels in both SLE and AAV despite only having genetic association with SLE. This correlation is present in both SLE and AAV three months following disease flare, but absent at the time of flare itself, perhaps because local inflammatory mechanisms provide a major contribution to sIL-2RA, overriding the influence of genotype. Thus the degree of autoimmune disease activity needs to be taken into account in studies attempting to correlate genotype with markers such as sIL-2RA.

There is precedent for such contrasting functional and genetic associations between SLE and AAV. FcγRIIIb, a neutrophil-specific low affinity activatory Fc receptor, has two allotypic variants, which have a significant effect on the affinity of the receptor for IgG. One of these, FCGR3B*NA1, is associated with SLE, and the other, FCGR3B*NA2, with AAV [20,21]. Moreover, copy number at *FCGR3B *is associated with expression and function of the receptor [22], and low copy number is associated with SLE [23] but not with AAV ([22] & data not shown). The association of a single gene with different diseases indicates the importance in disease of the biological pathway subserved by that gene, but it does not imply that defects in the gene lead to a similar functional perturbation in each case. Thus the same gene, *IL2RA*, is associated with both SLE and AAV, but may play quite distinct roles in these diseases.

## Conclusion

We confirm the association of the *IL2RA *locus with SLE, by genotyping rs11594656 across three independent cohorts, using two different study designs. We report evidence for a novel association of the *IL2RA *locus with AAV. The MS-associated SNP, rs2104286, is not associated with either disease in our cohorts. Soluble IL-2RA concentrations were elevated in SLE and AAV. After three months of treatment, soluble IL-2RA concentrations in both diseases correlated with the SLE associated rs11594656 genotype. The contrasting genetic and functional associations found between SLE and AAV and the *IL2RA *locus presumably reflect differences in disease pathogenesis.

## Competing interests

The authors declare that they have no competing interests.

## Authors' contributions

KGCS and PAL conceived and designed the study. AW, TJV, JAT, KL & RAW provided access to DNA samples, and TJV & JAT assisted in data interpretation. EJC performed the SNP genotyping and its analysis. Additional genotyping and experimental oversight was provided by CEL. MRC contributed the ELISA data and its analysis. The manuscript was written by EJC & KGCS. Additionally, MRC, CEL, TJV, PAL & JAT actively contributed to discussion of the results of this manuscript. All authors read and approved the final manuscript.

## Pre-publication history

The pre-publication history for this paper can be accessed here:


